# Supplementary material for: An integrated method for the leakage fault mode diagnosis and life prediction of the reactor coolant pump
Source: PLoS One. 2024 Jun 28;19(6):e0304652. doi: 10.1371/journal.pone.0304652 (PMC11213310; doi:10.1371/journal.pone.0304652)
Supplement: Attachment — Submitted filename: Marked corrections.pdf [file pone.0304652.s007.pdf]

PLOS ONE

An integrated method for leakage fault mode diagnosis and life prediction of the reactor coolant pump

--Manuscript Draft--

**Manuscript Number:**	PONE-D-24-02854
**Article Type:**	Research Article
**Full Title:**	An integrated method for leakage fault mode diagnosis and life prediction of the reactor coolant pump
**Short Title:**	An integrated method for leakage fault mode diagnosis and life prediction of the reactor coolant pump
**Corresponding Author:**	Jie Liu<br>Beihang University<br>Beijign, CHINA
**Keywords:**	reactor coolant pump; leakage fault mode diagnosis; life prediction; integrated model; cross-sectional area of the first seal; start point of degradation
**Abstract:**	Reactor Coolant Pump is a key equipment in nuclear power plant. If the leakage exceeds a certain threshold, it may cause reactor overheating and shutdown. The Reactor Coolant Pump leakage fault usually has two patterns—corrosion and scaling. Accurately and efficiently diagnosing the leakage fault mode as early as possible and predicting its remaining useful life are important for taking timely maintenance measures. In this paper, an integrated method is proposed. Firstly, the cross-sectional area of the first seal is extracted as a fault indicator. The motivation is that corrosion may cause the cross-sectional area becoming larger, and that scaling may bring a smaller cross-sectional area. Based on the fluid mechanics theory, an integrated model with several uncertain parameters is established between the cross-sectional area and the temperature and leakage at the inlet and outlet of the first seal. In the diagnosing process, a modified change-detection method is proposed for detecting start point of degradation. Then, the unknown parameters in the previous relation are estimated and the degrading data before the start point of degradation is used to carry out the leakage fault mode diagnosis. Secondly, a time series model of ARIMA is established to predict its remaining useful life based on the degrading data after the start point of degradation. At last, the leakage degrading data from six Reactor Coolant Pumps of a Nuclear Power Plant is used to carry out the leakage fault mode diagnosis and life prediction, which proves that the integrated method proposed is accurate and efficient.
**Order of Authors:**	Yinghua Shao, Ph.D
	Rui Kang
	Jingwen Fu
	Jie Liu
**Additional Information:**	
**Question**	**Response**
**Financial Disclosure**<br><br>Enter a financial disclosure statement that describes the sources of funding for the work included in this submission. Review the submission guidelines for detailed requirements. View published research articles from *PLOS ONE* for specific examples.	Yes

Powered by Editorial Manager® and ProduXion Manager® from Aries Systems Corporation

Notes

This statement is required for submission and **will appear in the published article** if the submission is accepted. Please make sure it is accurate.<br><br>**Funded studies**<br>Enter a statement with the following details:<br>• Initials of the authors who received each award<br>• Grant numbers awarded to each author<br>• The full name of each funder<br>• URL of each funder website<br>• Did the sponsors or funders play any role in the study design, data collection and analysis, decision to publish, or preparation of the manuscript?<br><br>Did you receive funding for this work?	
Please add funding details.<br>as follow-up to "**Financial Disclosure**<br><br>Enter a financial disclosure statement that describes the sources of funding for the work included in this submission. Review the submission guidelines for detailed requirements. View published research articles from *PLOS ONE* for specific examples.<br><br>This statement is required for submission and **will appear in the published article** if the submission is accepted. Please make sure it is accurate.<br><br>**Funded studies**<br>Enter a statement with the following details:<br>• Initials of the authors who received each award<br>• Grant numbers awarded to each author<br>• The full name of each funder<br>• URL of each funder website<br>• Did the sponsors or funders play any role in the study design, data collection and analysis, decision to publish, or preparation of the manuscript?<br><br>Did you receive funding for this work?"	Acknowledgement<br>This work was supported by National Natural Science Foundation of China (No.52005027) and National key Laboratory of Science and Technology on Reliability and Environmental Engineering.

Powered by Editorial Manager® and ProduXion Manager® from Aries Systems Corporation

Notes

Please select the country of your main research funder (please select carefully as in some cases this is used in fee calculation).<br>as follow-up to "**Financial Disclosure**<br><br>Enter a financial disclosure statement that describes the sources of funding for the work included in this submission. Review the submission guidelines for detailed requirements. View published research articles from *PLOS ONE* for specific examples.<br><br>This statement is required for submission and **will appear in the published article** if the submission is accepted. Please make sure it is accurate.<br><br>**Funded studies**<br>Enter a statement with the following details:<br>• Initials of the authors who received each award<br>• Grant numbers awarded to each author<br>• The full name of each funder<br>• URL of each funder website<br>• Did the sponsors or funders play any role in the study design, data collection and analysis, decision to publish, or preparation of the manuscript?<br><br>Did you receive funding for this work?"	CHINA - CN
**Competing Interests**<br><br>Use the instructions below to enter a competing interest statement for this submission. On behalf of all authors, disclose any competing interests that could be perceived to bias this work—acknowledging all financial support and any other relevant financial or non-financial competing interests.<br><br>This statement is required for submission and **will appear in the published article** if the submission is accepted. Please make sure it is accurate and that any funding	The authors have declared that no competing interests exist.

Powered by Editorial Manager® and ProduXion Manager® from Aries Systems Corporation

Notes

sources listed in your Funding Information later in the submission form are also declared in your Financial Disclosure statement.<br><br>View published research articles from *PLOS ONE* for specific examples.<br><br>**NO authors have competing interests**<br><br>Enter: *The authors have declared that no competing interests exist.*<br><br>**Authors with competing interests**<br><br>Enter competing interest details beginning with this statement:<br><br>*I have read the journal's policy and the authors of this manuscript have the following competing interests: [insert competing interests here]*<br><br>* typeset	
**Ethics Statement**<br><br>Enter an ethics statement for this submission. This statement is required if the study involved:<br><br>• Human participants<br>• Human specimens or tissue<br>• Vertebrate animals or cephalopods<br>• Vertebrate embryos or tissues<br>• Field research<br><br>Write "N/A" if the submission does not require an ethics statement.<br><br>General guidance is provided below. Consult the submission guidelines for detailed instructions. **Make sure that all information entered here is included in the Methods section of the manuscript.**	N/A

Powered by Editorial Manager® and ProduXion Manager® from Aries Systems Corporation

Notes

**Format for specific study types**<br><br>**Human Subject Research (involving human participants and/or tissue)**<br>• Give the name of the institutional review board or ethics committee that approved the study<br>• Include the approval number and/or a statement indicating approval of this research<br>• Indicate the form of consent obtained (written/oral) or the reason that consent was not obtained (e.g. the data were analyzed anonymously)<br><br>**Animal Research (involving vertebrate animals, embryos or tissues)**<br>• Provide the name of the Institutional Animal Care and Use Committee (IACUC) or other relevant ethics board that reviewed the study protocol, and indicate whether they approved this research or granted a formal waiver of ethical approval<br>• Include an approval number if one was obtained<br>• If the study involved *non-human primates*, add *additional details* about animal welfare and steps taken to ameliorate suffering<br>• If anesthesia, euthanasia, or any kind of animal sacrifice is part of the study, include briefly which substances and/or methods were applied<br><br>**Field Research**<br><br>Include the following details if this study involves the collection of plant, animal, or other materials from a natural setting:<br>• Field permit number<br>• Name of the institution or relevant body that granted permission	
**Data Availability**<br><br>Authors are required to make all data underlying the findings described fully available, without restriction, and from the time of publication. PLOS allows rare exceptions to address legal and ethical concerns. See the PLOS Data Policy and FAQ for detailed information.	Yes - all data are fully available without restriction

Powered by Editorial Manager® and ProduXion Manager® from Aries Systems Corporation

Notes

Manuscript
Click here to access/download;Manuscript;Manuscript.docx

An integrated method for leakage fault mode diagnosis and life prediction of the reactor coolant pump

Yinghua Shao[1], Rui Kang[2], Jingwen Fu[3], Jie Liu[4*]

[1] School of Reliability and Systems Engineering, Beihang University, Beijing, China

[2] School of Reliability and Systems Engineering, Beihang University, Beijing, China

[3] School of Reliability and Systems Engineering, Beihang University, Beijing, China

[4] School of Reliability and Systems Engineering, Beihang University, Beijing, China

* Corresponding author

E-mail: liujie805@buaa.edu.cn

1

Since the affiliation is the same you do not need to write it again and again. Just use the same number for all authors. In fact you do not even need numbers.

Notes

Abstract

Reactor Coolant Pump is a key equipment in nuclear power plant. If the leakage exceeds a certain threshold, it may cause reactor overheating and shutdown. The Reactor Coolant Pump leakage fault usually has two patterns—[1] corrosion and scaling. Accurately and efficiently diagnosing the leakage fault mode as early as possible and predicting its remaining useful life are important for taking timely maintenance measures. In this paper, an integrated method is proposed. Firstly, the cross-sectional area of the first seal is extracted as a fault indicator. The motivation is that corrosion may cause the cross-sectional area becoming larger, and that scaling may bring a smaller cross-sectional area. Based on the fluid mechanics theory, an integrated model with several uncertain parameters is established between the cross-sectional area and [2] the temperature and leakage at the inlet and outlet of the first seal. In the diagnosing process, a modified change-detection method is proposed for detecting start [3] point of degradation. Then, the unknown parameters in the previous relation are estimated and the degrading data before the start point of degradation is used to carry out the leakage fault mode diagnosis. Secondly, a time series model of ARIMA [4] is established to predict its remaining useful life based on the degrading data after the start point of degradation. At last, the leakage degrading data from six Reactor Coolant Pumps of a Nuclear Power Plant is used to carry out the leakage fault mode diagnosis and life prediction, which proves that the integrated method proposed is accurate and efficient.

Introduction

Nuclear power plants have been operating in nearly 30 countries to generate electricity, accounting for approximately 17% of the world's total electricity generation [1]. Nuclear energy has become one of the most important sources of energy. Although nuclear power plants in the world have rich operating experience and good safety records, due to the impact of nuclear pollution, the safety of nuclear reactors is still the most concerned issue in the development of nuclear energy [2].

The Reactor Coolant Pump (RCP) is one of the most important equipment in a Nuclear Power Plant (NPP) and is known as the heart of the reactor cooling system, and its safety is also crucial [3]. Therefore, effective fault diagnosis and health

1. problems
2. ,
3. the starting
4. Define ARIMA first

Notes

diagnosis and life prediction for the RCP of NPP, which can monitor the leakage during the operation of the nuclear main. From the point of meeting the needs of actual operation and maintenance, this method tries to combine the advantages of both the physical model diagnosis and the data-driven model diagnosis, using the quantity of flow from the output side of the primary pump as the monitoring indicator and using the cross-sectional area as the characteristic index, together to diagnose the pattern of failure happened to the seal and predict its remaining useful life. In this way, proper measures can be taken when the leakage quantity is abnormal, to ensure a healthy and stable operation of the pump and prevent unexpected shutdowns of nuclear power plants and serious accidents.

The remainder of the manuscript is structured as below. Section 2 introduces the structure of this integrated method, then introduced all the steps in this method in detail. In section 3, a case study is carried out to verify the effectiveness of the method. At last, a conclusion is made to evaluate the applicability and improvement direction of the method.

The Structure of the integrated method

Fig 2. The Structure of the integrated method.

The integrated method can be divided into three major steps. The first step is called Health Monitoring. In this step, it goes through all the leakage data and tries to find out the degradation point [28,29] as a dividing point, for which before the degradation point all the data can be seen as normal data, and after the degradation point can be seen as abnormal data. In this case, we assume that before the degradation point, the primary seal maintains normal operation and no fault mode occurs, so the data can be used to build the model and optimize the parameters. On the contrary, we assume that after the degradation point, failure occurs to the primary seal, so that all the data after the degradation point are used to diagnose the fault mode of the primary seal.

Fig 3. The process of the integrated method.

The second step is called fault mode diagnosis. In the process of fault diagnosis, it is mainly divided into five small steps:

(1) Physical model construction. We need to construct the physical relationship

Notes

This should be two and three together in figure 2

between the detection value and the characteristic value to analyze the fault model of the primary sealing device through the existing detection value data.

(2) Parameter magnitude analysis and optimization. In the obtained model, due to the unknown parameters, we need to optimize the parameters through data-driven means. While the model is based on physics instead of pure data-driven, each parameter has its actual physical meaning, so the magnitude of the parameter can be roughly deduced before the optimization process, in order to make the parameter of optimization more specific and to reduce the amount of calculation. Then python30 is used to optimize the unknown parameters in the equation obtained in step (1).

(3) Characteristic value calculation. Bring the parameters obtained in the previous step and the known detection values into equation obtained in step (1) to find the trend of the characteristic value.

(4) Trend judgment. Some methods are used to determine the trend of the obtained characteristic value curve to diagnose the fault type of the primary sealing device.

The third step is the remaining useful life prediction. First, the failure threshold of the monitored data is to be determined; Secondly, the degradation process is modeled with the monitoring data after the degradation point; Thirdly, the remaining useful life can be obtained by the failure threshold and degradation model.

Health Monitoring

Degradation Point Detection is one of the most important work in Health Monitoring. Degradation Point also called change point is the beginning of the change of monitoring status and the beginning of system failure. Detecting the degradation point is the revenge of fault diagnosis and prediction. N-point average value detection method is proposed in this paper to detect the degradation point.

Record all data points as $Q_1, Q_2, \ldots, Q_i, \ldots, Q_N$, for the N-point average method, for any integer $(\mathrm{i} \geq \mathrm{n}+1)$, let

$$\overline{Q_{\mathrm{i},\ n}} = \frac{x_i + x_{i+1} + \ldots + x_{i+n-1}}{n},$$

$$\overline{Q_{i-1,\ n}} = \frac{x_{i-n} + x_{i-n+1} + \ldots + x_{i-1}}{n},$$

requirements to be met in inequality (1):

$$\overline{Q_{i,n}} > a\overline{Q_{i-1,n}} \tag{1}$$

Also, requirements to be met in inequality (2):

Notes

are

$$Q_i > b\overline{Q_i} \quad (2)$$

a, b is two coefficients that can be determined or fitted based on the actual needs of health monitoring. Then this point is the point of leakage change, also known as the starting point of shaft seal degradation.

Fault mode Diagnosis

After the degradation point is detected, Fault mode Diagnosis can be carried out with the normal data before the degradation point.

Physical model construction

The major two fault modes for the mechanical seal include corrosion [31] and abrasion [32]. When corrosion occurs, the cross-sectional area of the seal will increase, while on the contrary, the cross-sectional area of the seal will decrease when abrasion happens [33]. In this way, we can use the cross-sectional area of the low channel as the fault characteristic quantity to indicate the fault mode of the mechanical seal, thereby providing help for the fault diagnosis when the leakage of the seal is abnormal.

In this article, we simplify the research of the primary mechanical seal shown in Fig 1 into the following model, as shown in Fig 4. And we simplified the coolant flowing through the sealing device into an ideal fluid model [34]. The ideal fluid model refers to a fluid model that ignores the effects of diffusion, viscosity, and heat conduction, and is also called an inviscid fluid [35]. The ideal fluid model can be divided into a compressible ideal fluid model [36] and an incompressible ideal fluid model [37] according to the compressibility. Here we choose the incompressible fluid model as the model used in this paper.

Fig 4. Simplified model of primary seal of nuclear pump.

According to Bernoulli's equation [38], for an ideal fluid to flow stably, at any point in the same flow tube, the sum of the kinetic energy and potential energy per unit volume of the fluid and the pressure at that point is a constant [39], as shown in Fig 5.

Fig 5. Bernoulli's equation of hydrodynamics.

$$p_1 + \frac{1}{2}\rho\upsilon_1^2 + \rho g h_1 = p_2 + \frac{1}{2}\rho\upsilon_2^2 + \rho g h_2 \quad (3)$$

$$p + \frac{1}{2}\rho v^2 + \rho g h = C \quad (4)$$

In equation (4) p is the pressure of a certain point in the fluid, v is the flow velocity of the fluid at that point, ρ is the fluid density, g is the acceleration of

Notes

needs correction

$$S_m = \frac{C_1 Q_m}{\sqrt{C_2 + C_3 Q_{in}^2 - C_4 Q_{out}^2}} \qquad (10)$$

Parameters C_1 , C_2 , C_3 , C_4 are introduced where

$$C_1 = \frac{1}{\mu}, C_2 = \frac{C_{out} - C_{in}}{\rho}, C_3 = \frac{1}{2S_{in}^2}, C_4 = \frac{1}{2S_{out}^2}.$$

In equation (10), S_m is the cross-sectional area of the pipeline of the primary seal device of the nuclear main pump, Q_m is the total quantity of coolant flowing through the pipeline, μ is a constant coefficient, C_{out} is a constant, C_{in} is a constant. Q_{in} is the quantity of coolant flowing through the input end of the nuclear main pump primary seal device, Q_{out} is the quantity of coolant flowing through the output end of the nuclear main pump primary seal device.

In actual working conditions of RCP, the temperature changes form the input end to the output end. Thus, the temperature changes is necessary to be considered in the model establishment process. From the Peng-Robinson equation [40], we can know that pressure is approximately proportional to temperature:

$$p = kT \qquad (11)$$

In equation (11), p is the pressure of the fluid, k is a constant coefficient, T is the temperature of the fluid. Combined by equation (10) and (11), the model that considers the temperature change conditions can be derived as follows:

$$S_m = \frac{Q_{out}}{\sqrt{C_1 \square T_{in} - C_2 \square T_{out} + C_3 \square T_{out} \square Q_{out}^2 \text{-} C_4 \square T_{in} \square Q_{in}^2}} \qquad (12)$$

1

In equation (12), T_{in} is the temperature of the Input End , and T_{out} is the temperature of the Output End. Therefore, the primary seal fault diagnosis model of the nuclear main pump can be simplified as Fig 6.

Fig 6. Simplified model of primary seal fault diagnosis of RCP.

According to the existing leakage data of six sets of pumps, we can use python to optimize the parameters to obtain specific values. It can further be calculated by bringing T_{in}, T_{out}, Q_{in}, Q_{out}, to calculate S_m. When S_m shows a growing trend and exceeds the set threshold, it means that the fault mode of the nuclear main pump is corrosion; when S_m shows a decreasing trend and exceeds the set threshold, it means

Notes

correction needed here

that the fault mode of the nuclear main pump is scaling.

Parameter magnitude analysis and optimization

According to the above derivation process, after the temperature standardization constant T_{ins} and T_{outs} is introduced, the expressions of the four parameters can be calculated as follows:

$$C_1 = \mu \cdot \sqrt{\frac{k}{\rho}} \cdot \frac{C_{in}}{T_{ins}} \tag{13}$$

$$C_2 = \mu \cdot \sqrt{\frac{k}{\rho}} \cdot \frac{C_{out}}{T_{outs}} \tag{14}$$

$$C_3 = \mu \cdot \sqrt{\frac{k}{\rho}} \cdot \frac{\rho}{2T_{outs} \cdot S_{out}{}^2} \tag{15}$$

$$C_4 = \mu \cdot \sqrt{\frac{k}{\rho}} \cdot \frac{\rho}{2T_{ins} \cdot S_{in}{}^2} \tag{16}$$

According to Bernoulli's theorem, the constant coefficients at the output and input are roughly the same:

$$p_{in} + \frac{1}{2}\rho v_{in}{}^2 \approx p_{out} + \frac{1}{2}\rho v_{out}{}^2 \tag{17}$$

In this way, we can get: $C_{in} \approx C_{out}$.

Since $T_{ins} \approx T_{outs}$, we can get: $C_1 \approx C_2$.

Because the cross-sectional area at the input end is larger than output end, S_{in} [illegible] S_{out}

1

Taking the experience value $S_{in} = 10S_{out}$, we can get: C_3 [illegible] C_4.

$C_4 = 1$ is supposed, we can get $C_3 = 100$, and the connection between C_2 and C_3 can be illustrated as follows:

$$\frac{C_2}{C_3} = \frac{2C_{out}S_{out}{}^2}{\rho} = \frac{2C_{out}}{\rho}\left(\frac{Q_{out}}{v_{out}}\right)^2 \tag{18}$$

In the actual fault diagnosis process, C_1、C_2、C_3、C_4 will be taken into python for parameter optimization to obtain specific values using the on-site monitoring data of mechanical seal leakage of RCP.

Characteristic value calculation

In this step, C_1、C_2、C_3、C_4 calculated from last step and $T_{in}, T_{out}, Q_{in}, Q_{out}$ from on-site detection will be taken into equation (12) to obtain a specific value of S_m. Then a

Notes

in the following table 7.

Table 7 .Failure threshold of primary seal leakage of main pump in nuclear power plant.

Pump No.	The degradation point	Leakage(l/h)	Change trend	Threshold(l/h)
**1**	1929	241	Rise	289
**2**	2006	262	Rise	314
**3**	636	236	Descend	197
**4**	947	241	Rise	289
**5**	452	254	Rise	305
**6**	449	258	Rise	310

Degradation process modeling based on ARIMA model

According to the leakage data after the degradation point of six nuclear main pumps, we established a prediction model based on ARIMA with the help of Python programming, as shown in Fig 12. and Fig 13. Fig 11. is the ARIMA model applicable to short-term prediction. It forecasts 6 points with high prediction accuracy. Fig 12. is the ARIMA model applicable to long-term prediction. It can predict the long-term change trend by predicting 1000 points. According to the "mse" value, ARIMA model can model the degradation process with better leakage data, whether it is short-term prediction or long-term prediction.

Fig 12. ARIMA model for short-term prediction. 1

Fig 13. ARIMA model for long-term prediction. 2

Remaining useful life prediction

According to ARIMA model, the corresponding failure point when the leakage threshold is reached for the first time can be obtained. The time difference between the failure point and the observation point is the remaining service life. The solution results of the remaining service life of the primary seal of the main pump in six nuclear power plants are shown in Table 8.

Table 8. Remaining useful life of primary seal leakage of main pump in nuclear

cannot follow anything from the figure

cannot follow anything from the figure

Notes

Conclusions

power plant

Pump No.	The degradation point	Leakage (l/h)	Change trend	Threshold (l/h)	Failure point	RUL (h)
**1**	1929	241	Rise	289	1971	178
**2**	2006	262	Rise	314	2046	170
**3**	636	236	Descend	197	908	1155
**4**	947	241	Rise	289	1162	913
**5**	452	254	Rise	305	485	140
**6**	449	258	Rise	310	504	234

Conclusion

1

This paper proposes a new method for the leakage pattern diagnostics in reactor coolant pump, combining the advantages from the physical model and data-driven method. This method is both simple to implement and has low calculation complexity. Moreover, it reveals the relationship between the detectable value Q_{out} and the undetectable characteristic value S_m, so that the changing trend of S_m can be inferred and used for further maintenance measures to be taken.

However, this method still has certain limitations. First, in the derivation of the physical model, we assumed that the model is ideal fluid with horizontal movement. But in practical engineering application, there can be problems such as height difference or heat exchange, which may impact on the accuracy of the model. Second, the so-called actual degradation points given in this case are actually given by experts based on their experience. In our degradation point detection process, we pursue the calculated value as close to the real degradation point as possible, but there may be some human error from the beginning.

In future research, we hope to further integrate this method with the nuclear power plant operation and maintenance process, to conduct inspection and prediction of the

Notes

Figure

Click here to access/download;Figure;Fig 1. The Reactor Coolant Pump of the Nuclear Power Plant.tif

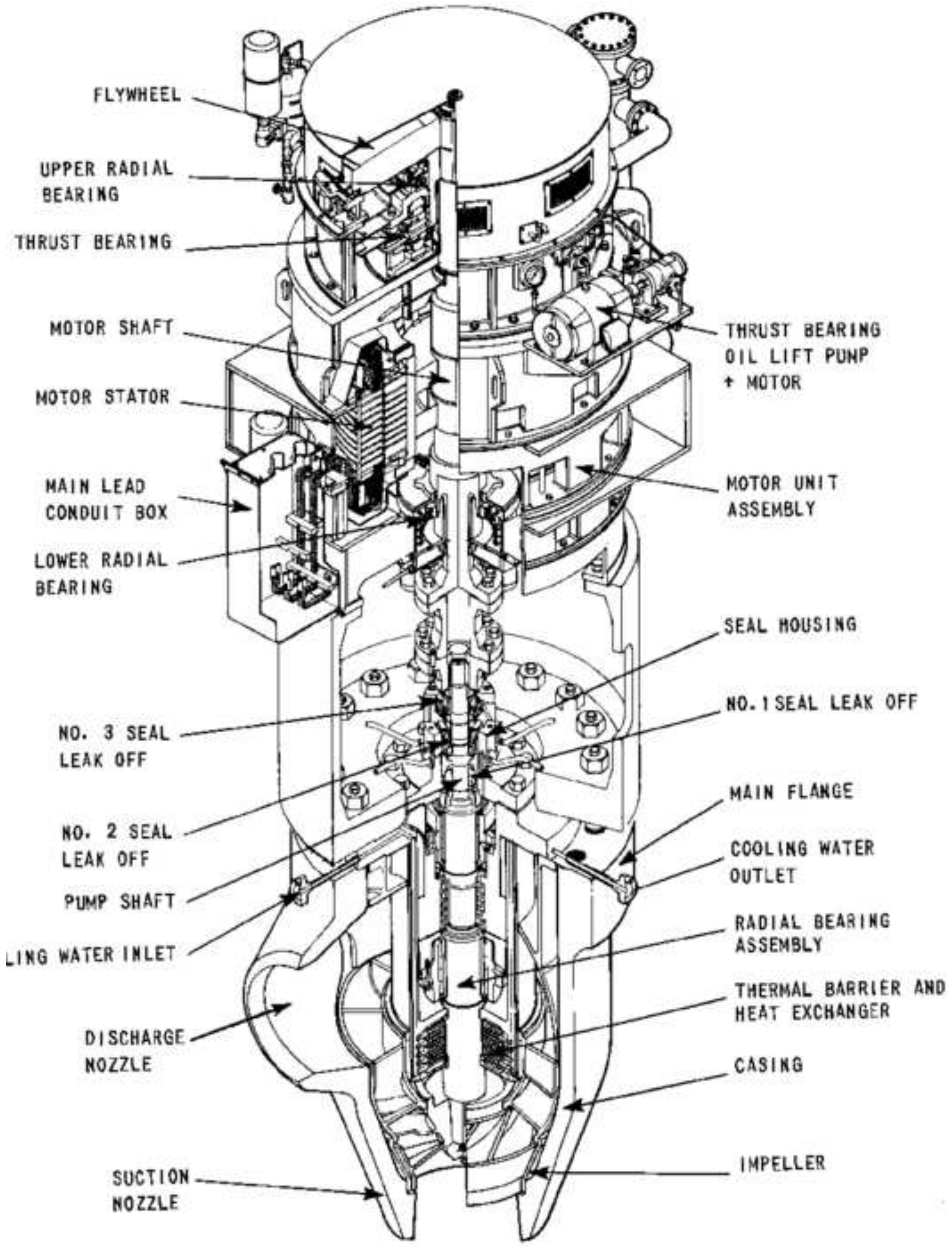

Notes

Figure

Click here to access/download;Figure;Fig 2. The Structure of the integrated method.tif

merge magnitude analysis and optimization

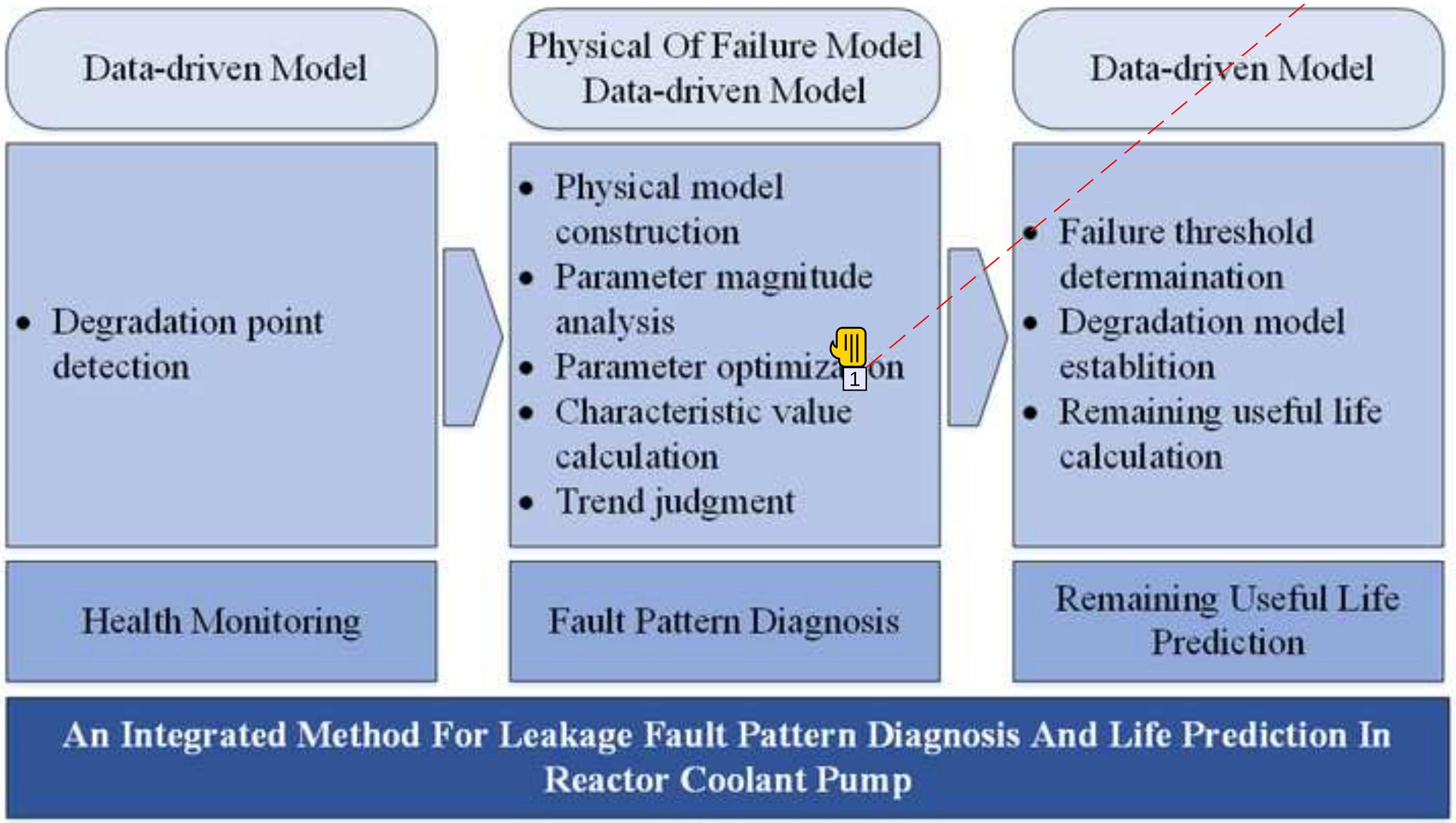

Notes

Figure

Click here to access/download;Figure;Fig 3. The process of the integrated method.tif

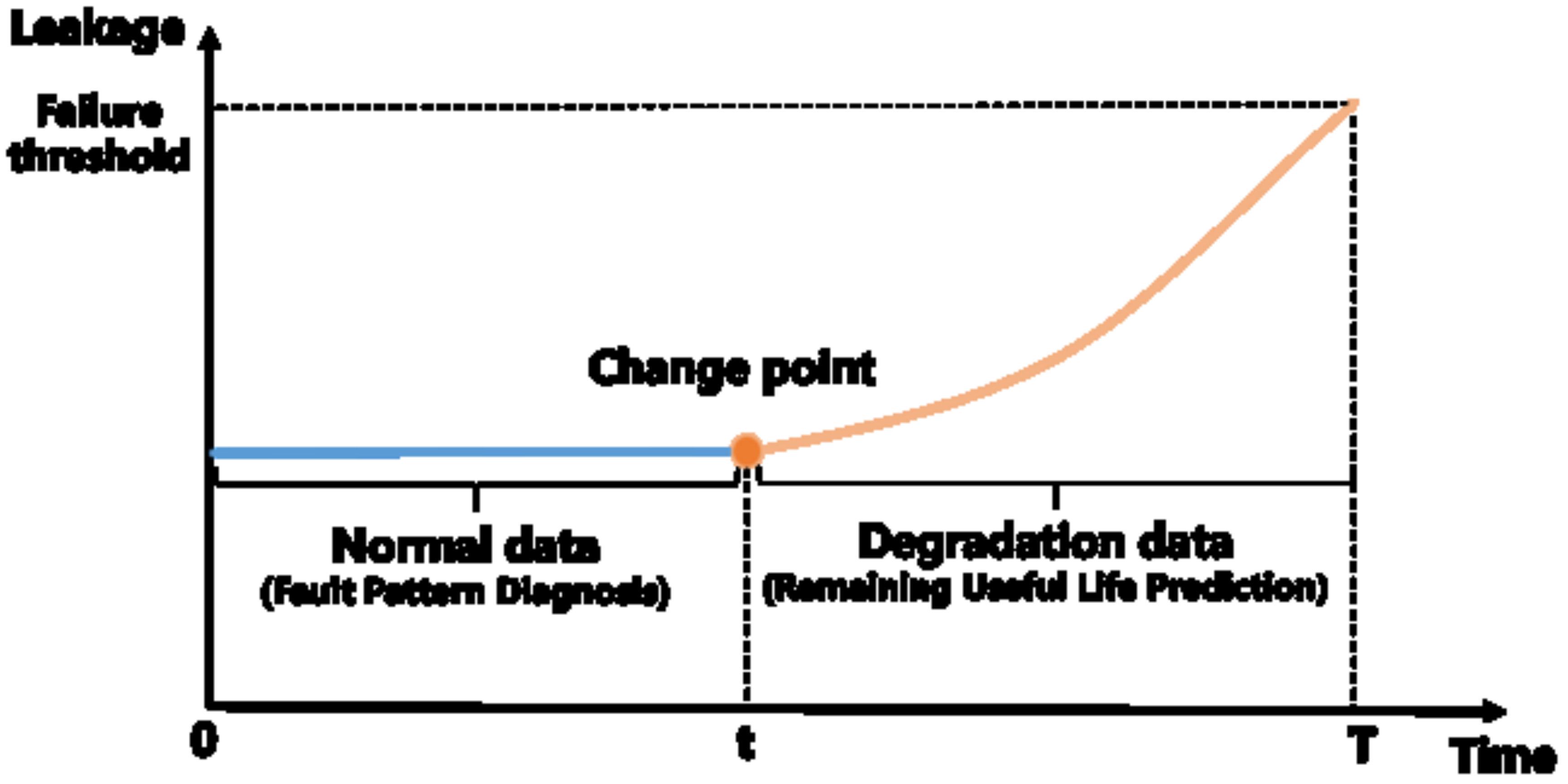

Notes

Figure

Click here to access/download;Figure;Fig 4. Simplified model of primary seal of nuclear pump.tif

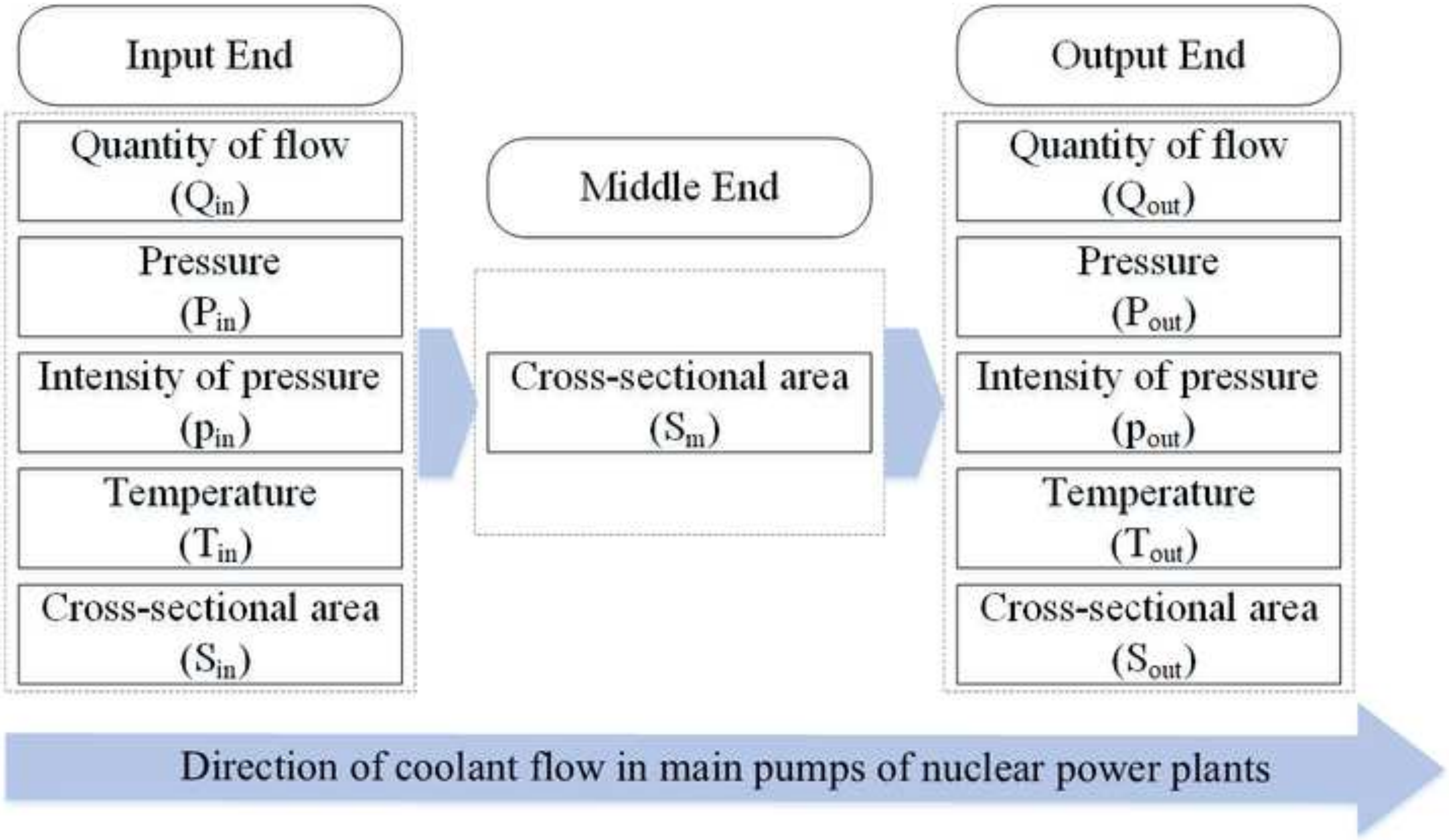

Notes

Figure

Click here to access/download;Figure;Fig 5. Bernoullis equation of hydrodynamics.tif

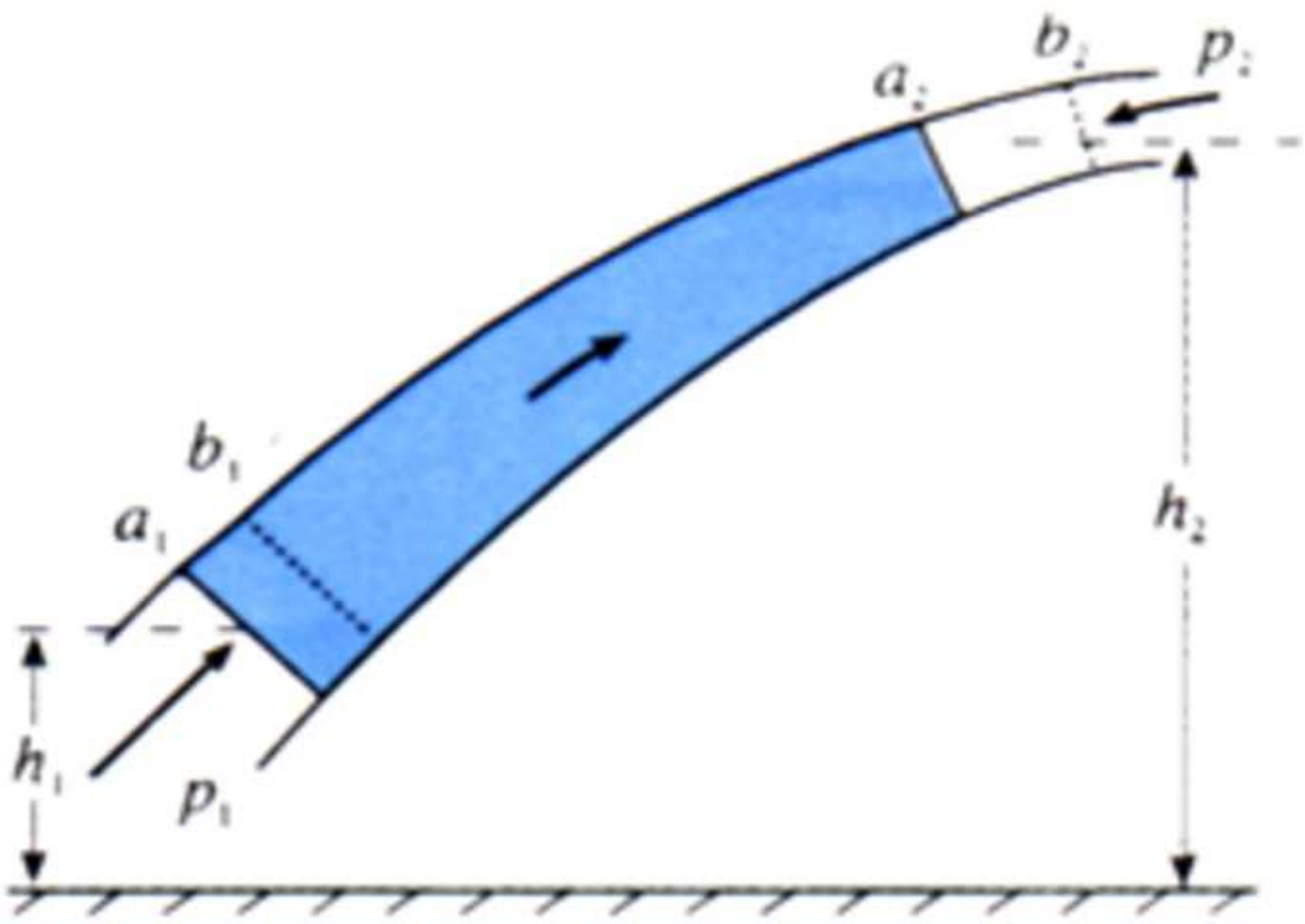

Notes

Figure

Click here to access/download;Figure;Fig 6. Simplified model of primary seal of RCP.tif

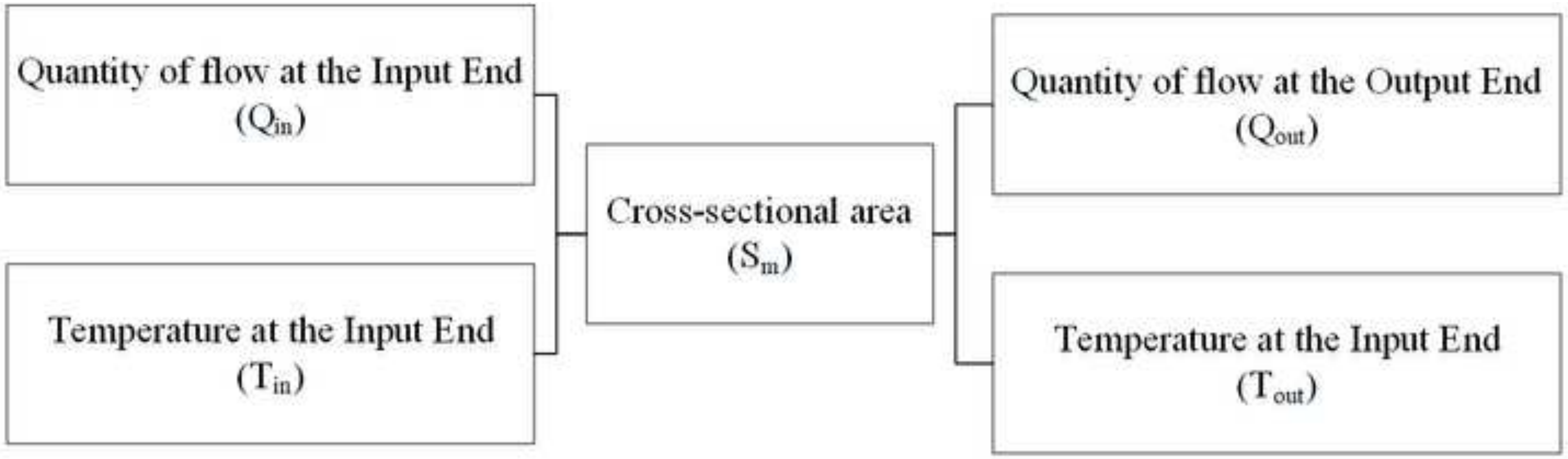

Notes

Figure

Click here to access/download;Figure;Fig 7. Procedure for establishing ARIMA model.tif

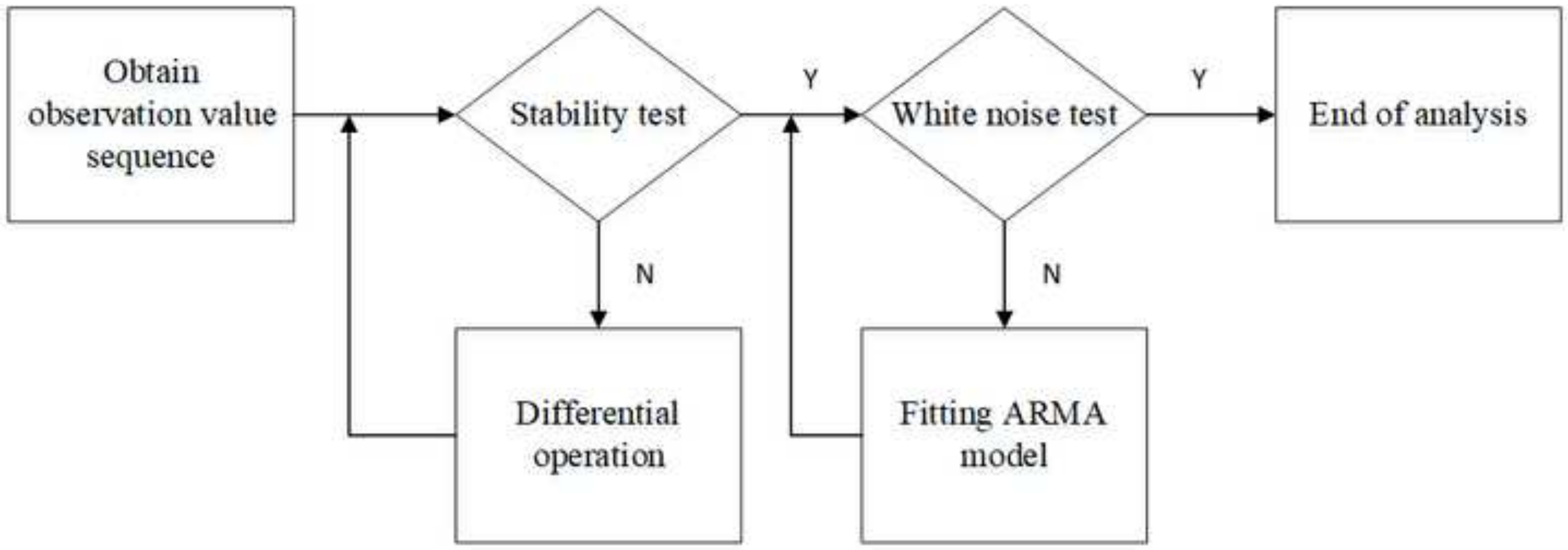

Notes

Figure

Click here to access/download;Figure;Fig 8. Temperature at input and output end.tif

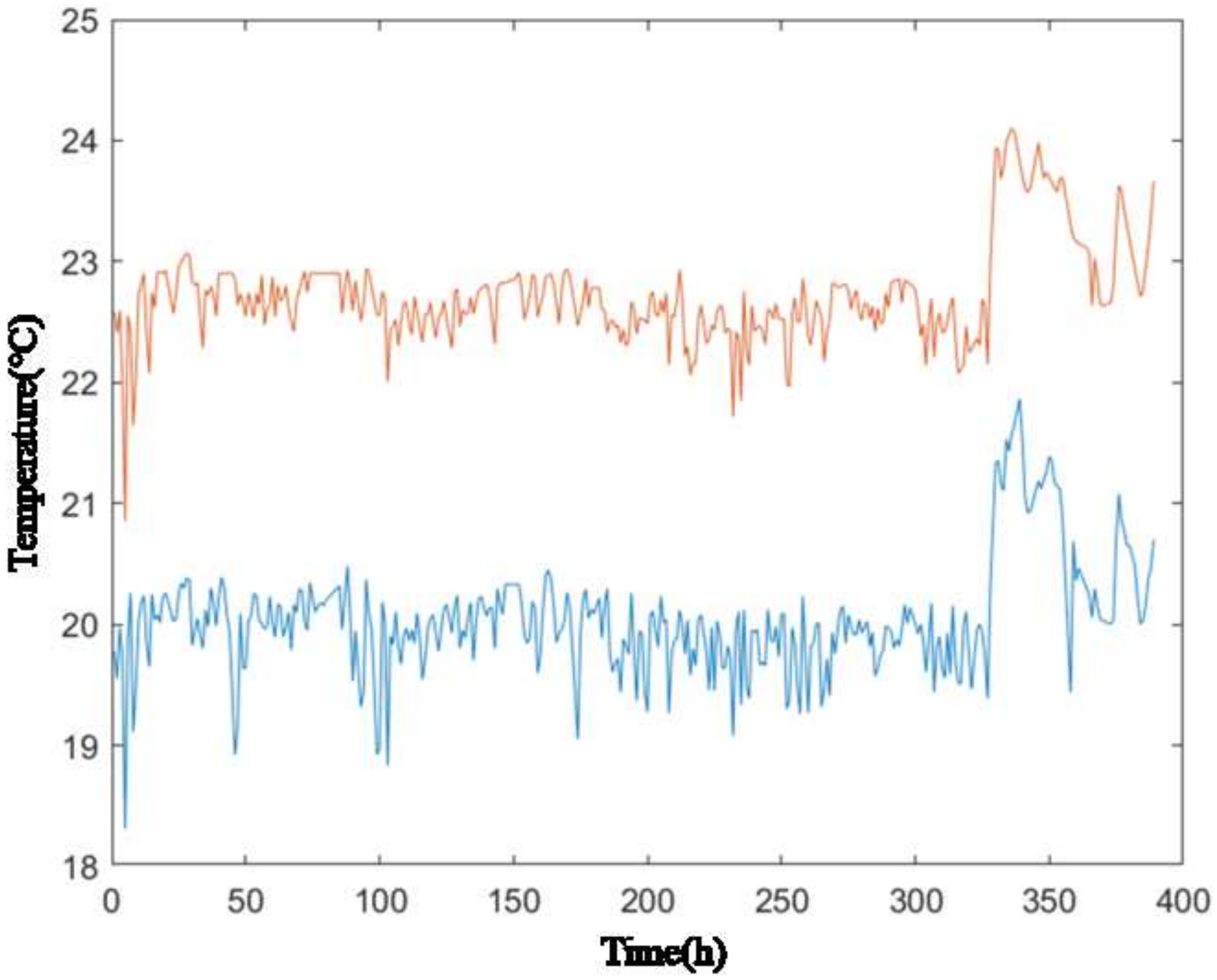

Notes

Figure

Click here to access/download;Figure;Fig 9. Leakage at input and output end.tif

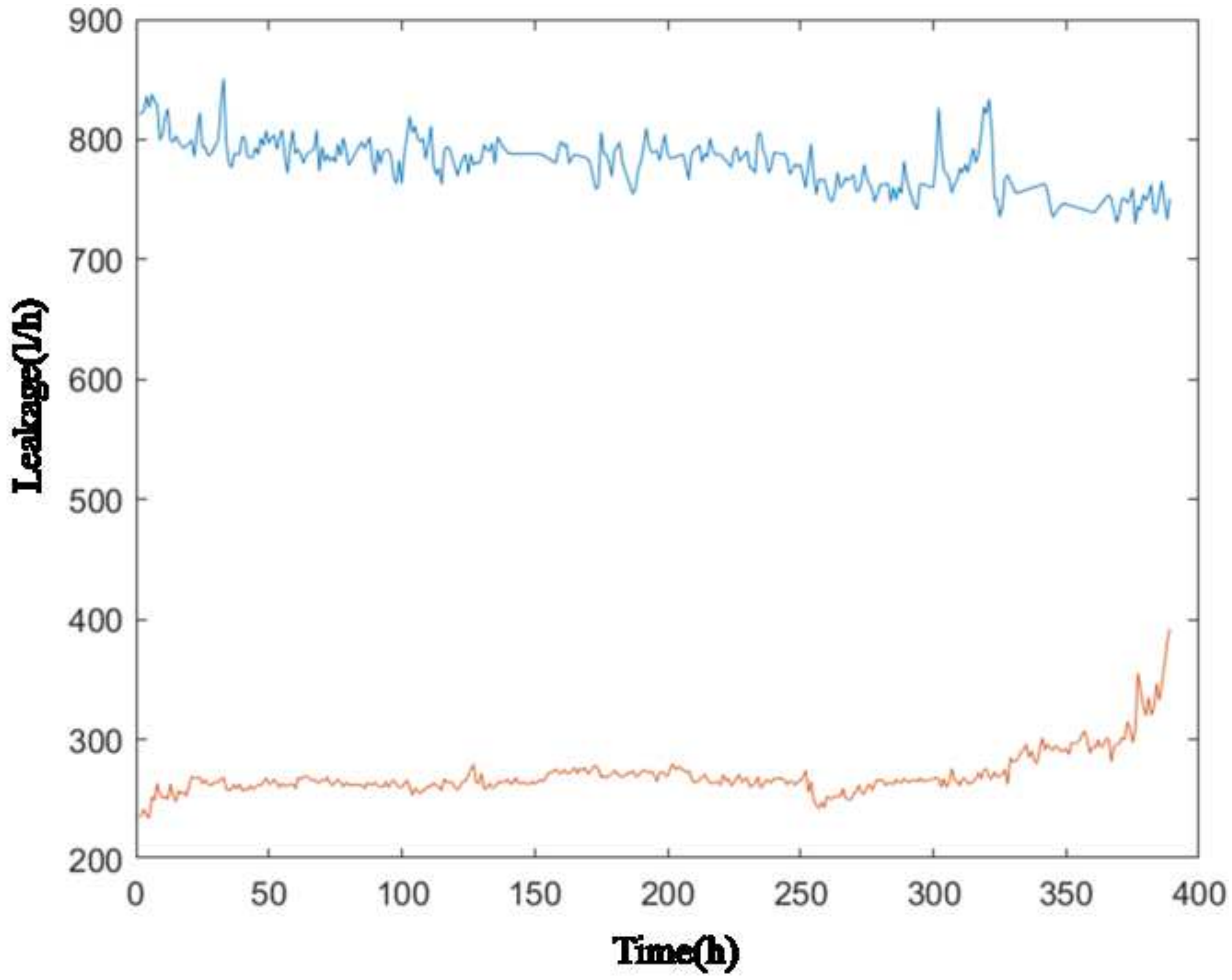

Notes

Figure

Click here to access/download;Figure;Fig 10. When n=55, solve the optimal value image.tif

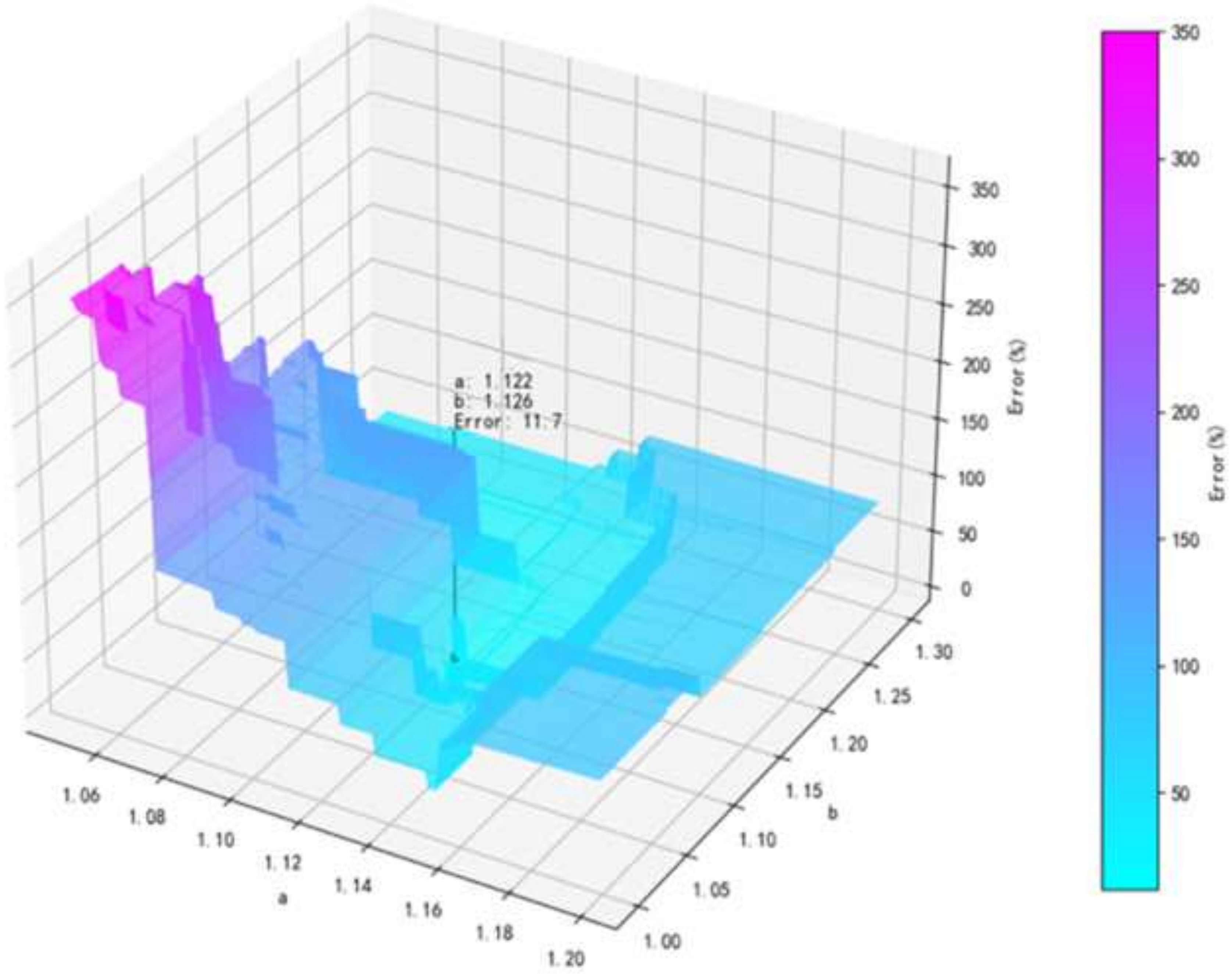

Notes

Figure

Click here to access/download;Figure;Fig 11. Curve obtained after parameter optimization.tif

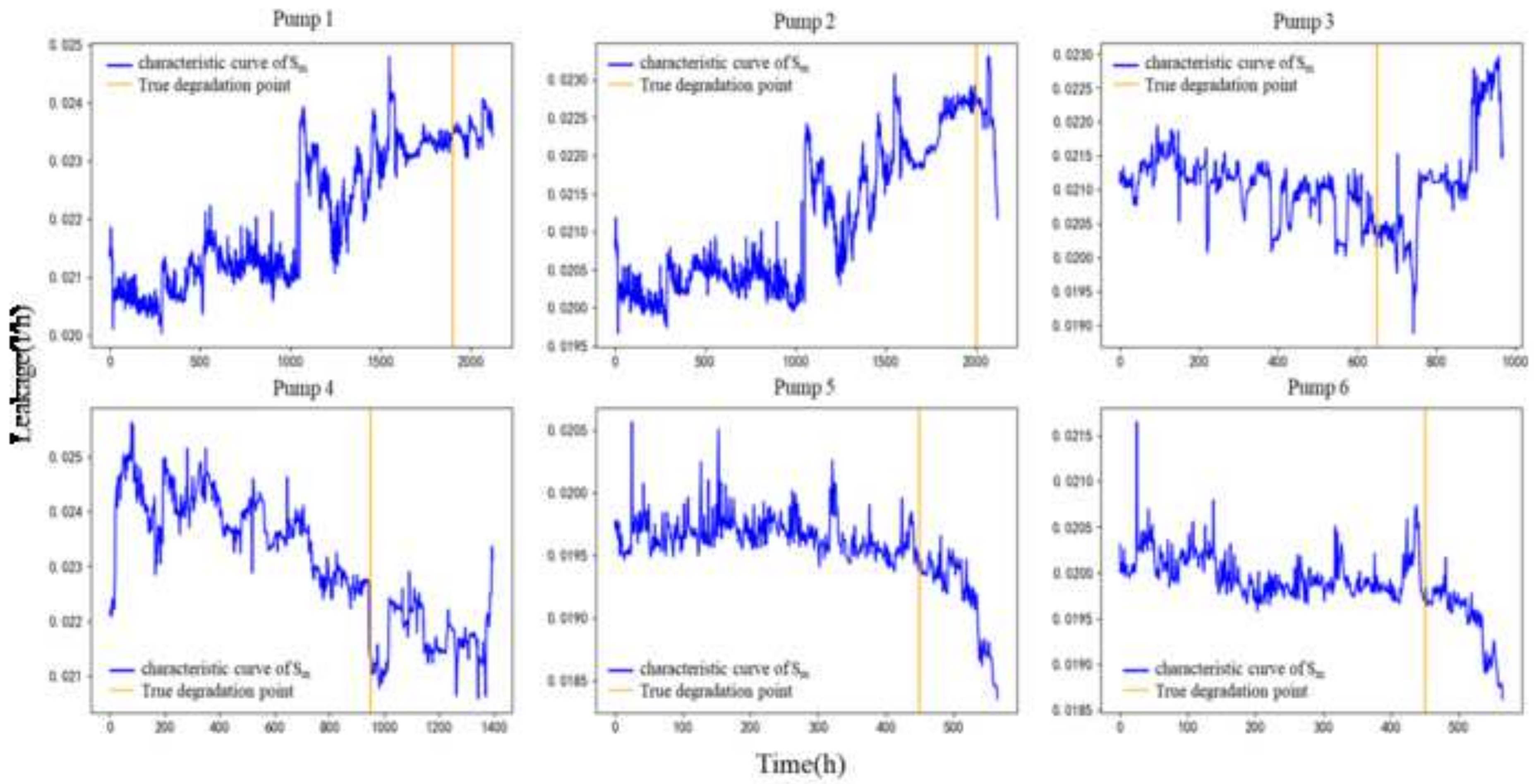

Notes

Figure

Click here to access/download;Figure;Fig 12. ARIMA model for short-term prediction.tif

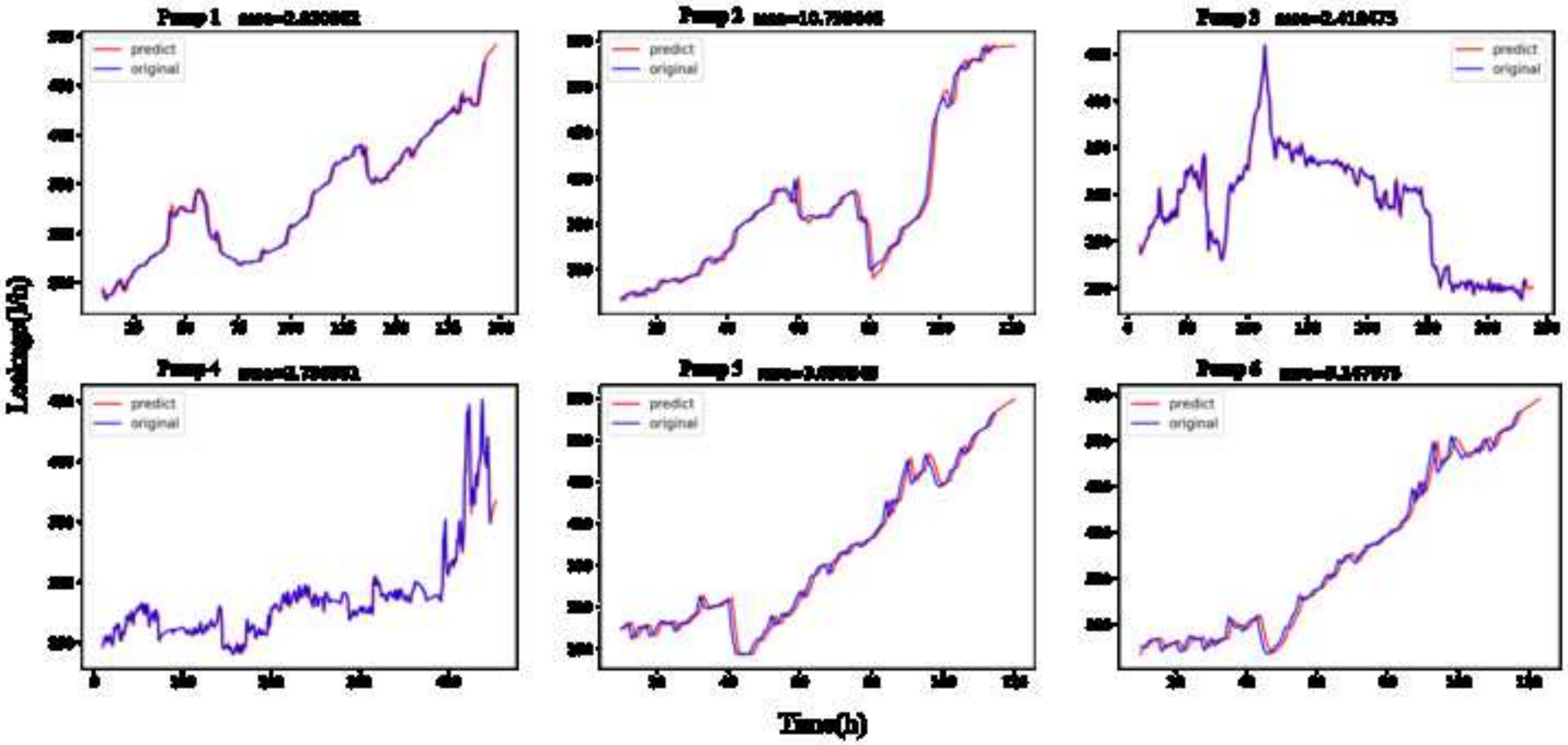

Notes

Figure

Click here to access/download;Figure;Fig 13. ARIMA model for long-term prediction.tif

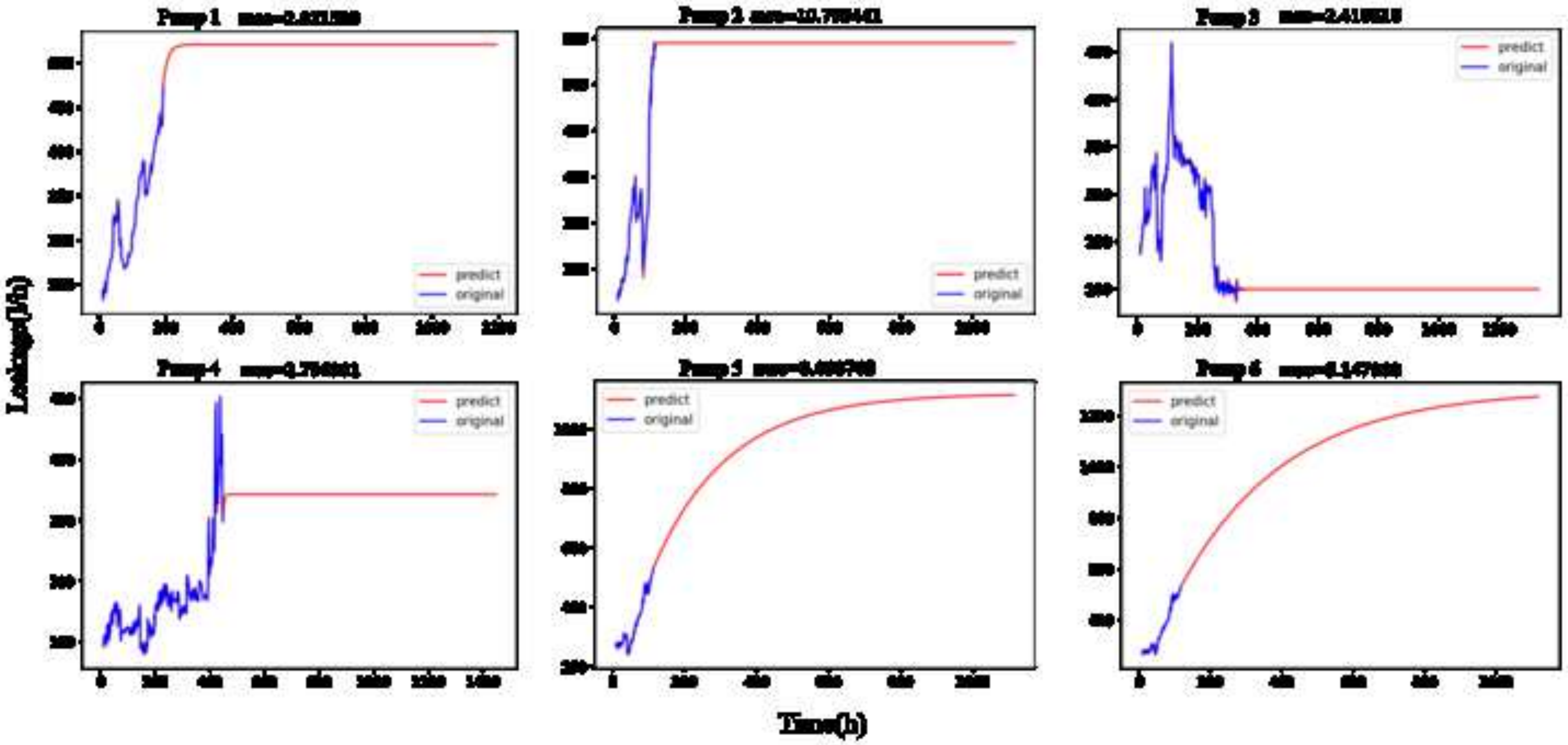

Notes
