# Supplementary material for: An integrated method for the leakage fault mode diagnosis and life prediction of the reactor coolant pump
Source: PLoS One. 2024 Jun 28;19(6):e0304652. doi: 10.1371/journal.pone.0304652 (PMC11213310; doi:10.1371/journal.pone.0304652)
Supplement: Attachment — Submitted filename: Response to Reviewers.docx [file pone.0304652.s008.docx]

**Response to Reviewers**

**Reviewer #1: The authors presented new method for the leakage pattern diagnostic in reactor coolant pump. They compared the degradation values obtained from their models with the real degradation data, however there are big errors appeared, the authors requested to explain more their model and the associated results, some other comments are the following**

**Answer:** The model and associated results have been explained more clearly. With the degradation point detection error rates not exceeding 4%, fault mode diagnosis correction rates 100% and practical RUL predicting results, the integrated method proposed is proved to be accurate, efficient and practical.

**1- All figures need to be modified for better quality, the figures that shown in the manuscript are not good to be presented in term of quality**

**Answer:** All figures have been compiled and saved as a tiff file with LZW compression using Photoshop software to be presented in term of quality.

**2- Figure 8 and Figure 9 don’t have legend, what are the blue and orange lines**

**Answer:** The legends have been added to Figure 8 and Figure 9.

**3- You need to explain more about the results shown in Figure 10. For example, why at n=55 the error is the lowest, and what is the reason of higher errors..etc. please explain your results in more details**

**Answer:** The N-point average value detection method proposed in this paper is used to detect the degradation point in order to eliminate the impact of outliers produced by environmental, working conditions and other interference factors on monitoring data during the health monitoring process. In this method, there are three parameters a, b, and n, that need to be optimized. The constraint objective of optimization is to minimize the error between the obtained degradation point and the true degradation point in Table 1. Subsequently, this article uses Python programs to optimize and finally obtain the optimal value of the three parameters, which is n=55, a=1.122, b=1.126, and at this point, the minimum error rate is 11.7%. Used the 55-piont average value detection method with parameters a=1.122, b=1.126, the degradation points of six pumps can be determined in Table 3. with error rates not exceeding 4% which is proved to be effective.

**4- The results that shown in Table 6 need to be explained in more details.**

**Answer:** The results that shown in Table 6 have been explained in more details: The trend detection of the curves of $S_{m}$ changing with time in Fig 11. is carried out using the Cox-Stuart detection method. Under Cox-Stuart detection method, when$S_{+}<S_{-}$, the trend of $S_{m}$ is rising; while$S_{+}>S_{-}$ the trend of $S_{m}$ is Descending. The trend judgement results can be obtained in Table 6.

**5- What is the degradation point in more details**

**Answer:** Degradation Point also called change point is the beginning of the change of monitoring status and the beginning of system failure. Detecting the degradation point is the revenge of fault diagnosis and prediction. The degradation point is illustrated in Fig 3.

**6- In page 24, there is a shifting in figure caption**

**Answer:** The figure caption in page 24 is corrected as Fig 10. When n=55, solve the optimal value image to maintain uniformity.

**7- It is very difficult to read figure 12 and 13, and many other figures**

**Answer:** All figures have been compiled and saved as a tiff file with LZW compression using Photoshop software to be presented in term of quality.

**Reviewer #2: There are too many typos in the paper and the figures are mostly not legible. The procedure is also all based on derived analytical correlations. Please see the marked comments and corrections in the manuscript.**

**Answer:** The words, figures and analysis have been reorganized following the marked comments and corrections in the manuscript.

**Reviewer #3:**

**- The abstract does not include sufficient quantitative data. Please revise it.**

**Answer:** Quantitative data has been added to the abstract to illustrate the work in the paper more clear and practical: “At last, the leakage degrading data from six Reactor Coolant Pumps of a Nuclear Power Plant is used to carry out the leakage fault mode diagnosis and life prediction with degradation point detection error rates not exceeding 4%, fault mode diagnosis correction rates 100% and practical RUL predicting results, which proves that the integrated method proposed is accurate and efficient.”

**- The Abstract should contain answers to the following questions: What problem was studied and why is it important? What methods were used? What are the important results? What conclusions can be drawn from the results? What is the novelty of the work and where does it go beyond previous efforts in the literature? Please include specific and quantitative results in your Abstract, while ensuring that it is suitable for a broad audience.**

**Answer:** The Abstract is reorganized more clear and meaningful.

“Reactor Coolant Pump is a key equipment in nuclear power plant. If the leakage exceeds a certain threshold, it may cause reactor overheating and shutdown. The Reactor Coolant Pump leakage fault usually has two problems—corrosion and scaling. Accurately and efficiently diagnosing the leakage fault mode as early as possible and predicting its remaining useful life are important for taking timely maintenance measures. In this paper, an integrated method is proposed. Firstly, the cross-sectional area of the first seal is extracted as a fault indicator. The motivation is that corrosion may cause the cross-sectional area becoming larger, and that scaling may bring a smaller cross-sectional area. Based on the fluid mechanics theory, an integrated model with several uncertain parameters is established between the cross-sectional area, and the temperature and leakage at the inlet and outlet of the first seal. In the diagnosing process, a modified degradation-detection method is proposed for detecting the starting point of degradation. Then, the unknown parameters in the previous relation are estimated and the degrading data before the starting point of degradation is used to carry out the leakage fault mode diagnosis. Secondly, a time series model of Autoregressive Integrated Moving Average (ARIMA) is established to predict its remaining useful life based on the degrading data after the starting point of degradation. At last, the leakage degrading data from six Reactor Coolant Pumps of a Nuclear Power Plant is used to carry out the leakage fault mode diagnosis and life prediction with degradation point detection error rates not exceeding 4%, fault mode diagnosis correction rates 100% and practical RUL predicting results, which proves that the integrated method proposed is accurate and efficient. The integrated method proposed in this paper combines the advantages of both the physical model diagnosis and the data-driven model diagnosis and innovatively make up of the quantity of flow from the output side of the primary pump as the monitoring indicator and the cross-sectional area as the characteristic index together to diagnose the pattern of failure happened to the seal and predict its remaining useful life, which can meet the needs of actual operation and maintenance to ensure a healthy and stable operation of the pump and prevent unexpected shutdowns of nuclear power plants and serious accidents.”

**- In literature review, the authors should add more recent publications discussed the current used numerical methods to study TH analysis in nuclear power plants' cores such as Computational fluid dynamics (CFD) and other system codes. Why the ARIMA is selected among other codes and numerical methods? Authors are suggested to enhance the literature review with a section regarding the previous used numerical analyses for similar TH studies. Authors are recommended to modify the literature to include this discussion by adding below refs, which are relevant:**

**•https://doi.org/10.1063/5.0109255**

**•https://doi.org/10.1155/2019/4375782**

**• https://doi.org/10.1016/j.ijheatfluidflow.2023.109115**

**Answer:** TH analysis in nuclear power plants can be classified as the method based on fault physical models combined with simulation. And relative literature is adding to the reviews. The advantage of the integrated method proposed is the combination of the fault physical model and the data-driven model to carry out the diagnosis and prediction. The fault physical model based on the fluid mechanics theory is practical for the diagnosis of the nuclear plant and the ARIMA model is convenient and feasible in the prediction of the RUL of the nuclear plant.

**-The originality of the paper needs to be stated clearly. It is of importance to have sufficient results to justify the novelty of a high-quality journal paper. The Introduction should make a compelling case for why the study is useful along with a clear statement of its novelty or originality by providing relevant information and providing answers to basic questions such as: What is already known in the open literature? What is missing (i.e., research gaps)? What needs to be done, why and how? Clear statements of the novelty of the work should also appear briefly in the Abstract and Conclusions sections.**

**Answer:** The Introduction has been reorganized: First, the method based on fault physical models is reviewed including cumulative damage, warning devices and specific models such as TH analysis etc.; Then the method based on the data-driven model is reviewed including statistical regression, similarity, stochastic process etc. Both of the two methods above have disadvantages: the establishment of fault physical model requires a deep understanding of the fault evolution mechanism of the object, and is not suitable for prediction modeling of complex, non-linear, and uncertain objects; Due to the lack of explanation of the fault mechanism, the interpretability of the model is not strong, and long-term analysis and prediction cannot be carried out based on the data-driven model. And the integrated method proposed combines the advantages of both the physical model diagnosis and the data-driven model diagnosis and innovatively make up of the quantity of flow from the output side of the primary pump as the monitoring indicator and the cross-sectional area as the characteristic index together to diagnose the pattern of failure happened to the seal and predict its remaining useful life, which can meet the needs of actual operation and maintenance to ensure a healthy and stable operation of the pump and prevent unexpected shutdowns of nuclear power plants and serious accidents. And the novelty of the work is added to the Abstract and Conclusions.

**-An updated and complete literature review should be conducted and should appear as part of the Introduction. The results and findings should be compared to and discussed in the context of earlier work in the literature.**

**Answer:** The literature review has been reorganized illustrated in the answer above.

**- Is the constructed model's predictions validated against previous experimental and/or numerical data? Please explain.**

**Answer:** Yes. The validation of the integrated method on diagnosis and prediction is carried out in the Case part based on the leakage data, actual degradation point and actual fault pattern of six reactor coolant pumps shown in Table 1, Table 3, Table 6 , Table 7 and Table 8. With the degradation point detection error rates not exceeding 4%, fault mode diagnosis correction rates 100% and practical RUL predicting results, the integrated method proposed is proved to be accurate, efficient and practical.

**- Conclusion section sound very general. Please support by more quantitative data.**

**Answer:** The Conclusion section has been reorganized adding quantitative data to support the valuation of the work in this paper: With the degradation point detection error rates not exceeding 4%, fault mode diagnosis correction rates 100% and practical RUL predicting results, the integrated method proposed is proved to be accurate, efficient and practical.

**- Please increase the image quality for all figures in the manuscript.**

**Answer:** All figures have been compiled and saved as a tiff file with LZW compression using Photoshop software to be presented in term of quality.

- check the whole manuscript for grammar and typos.

**Answer:** The grammar and typos of the manuscript has been checked again.
